# Sagittal Long-Axis Bladder Diameter and CT-Measured Bladder Wall Thickness in Healthy Female Patients

**DOI:** 10.7759/cureus.116549

**Published:** 2026-09-19

**Authors:** Gavin L Feinberg, Celine Torkzad, Martha K Terris, Pardeep K Mittal, Michael R Torkzad

**Affiliations:** 1 Department of Biological Sciences, Augusta University, Augusta, USA; 2 Department of Science, St. Johns Country Day School, Orange Park, USA; 3 Department of Urology, Medical College of Georgia, Augusta University, Augusta, USA; 4 Division of Abdominal and Pelvic Radiology, Department of Radiology and Imaging, Medical College of Georgia, Wellstar MCG Health, Augusta University, Augusta, USA

**Keywords:** bladder distension, bladder wall thickness, computed tomography, pelvic imaging, urinary bladder

## Abstract

Objective: The objective of this study is to evaluate the association between CT-measured bladder wall thickness (BWT) and sagittal long-axis bladder diameter (SL), defined as the largest midsagittal luminal bladder diameter, in female patients without documented bladder disease or urinary symptoms, using SL as a practical indicator of bladder distension.

Methods: This retrospective study included 111 patients aged 16 years or older. An experienced abdominal radiologist measured BWT perpendicular to the anterior wall at the contact point of the SL measurement. Analyses included five descriptive SL categories, analysis of variance with Tukey testing, Pearson correlation, and linear and log-log regression. Post hoc age-adjusted models used heteroscedasticity-consistent type 3 (HC3) standard errors; sensitivity analyses examined an age cutoff of 18 years and omission of the largest SL observation.

Results: Mean BWT was 3.50 mm and mean SL was 62.05 mm. SL was inversely correlated with BWT (r = −0.51; P < 0.001). Mean BWT decreased from 6.00 mm in the 10-<30-mm category to 1.90 mm in the ≥90-mm category (F = 11.26; P < 0.001). Age was not significantly correlated with BWT (r = 0.035; P = 0.714). After age adjustment, the SL association persisted (coefficient, −0.0398 mm/mm; 95% CI, −0.0604 to −0.0193; P < 0.001), whereas the age coefficient was not significant (P = 0.528). Both sensitivity analyses retained the inverse SL-BWT association.

Conclusion: CT-measured BWT decreased as SL increased in female patients. Building on prior evidence relating SL to bladder volume, these findings support recording SL alongside BWT to describe wall thickness in relation to bladder distension. Reader reproducibility and diagnostic usefulness require further evaluation; the descriptive categories do not establish clinical thresholds.

## Introduction

Bladder wall thickness (BWT) is an increasingly recognized imaging parameter in the evaluation of bladder dysfunction, inflammation, obstruction, and neoplasia. In the urologic literature, BWT measured by ultrasound has been investigated as a noninvasive biomarker for detrusor overactivity, bladder outlet obstruction, and lower urinary tract symptoms [1]. This work has consistently shown that BWT is influenced by bladder filling, prompting the International Consultation on Incontinence-Research Society (ICI-RS) to emphasize the need for standardized measurement conditions and documentation of bladder distension at the time of measurement [2].

Classic ultrasound studies demonstrate the physiologic basis for this relationship. Jequier and Rousseau showed that BWT decreases as the bladder fills in children, while Hakenberg et al. reported a weak but statistically significant inverse relationship between bladder volume and BWT in healthy adults [3,4]. These findings are consistent with the bladder’s normal response to underdistension. When the bladder is incompletely filled, the wall remains relatively unstretched and mucosal folds may remain prominent, creating the appearance of wall thickening even in the absence of disease.

Despite this body of ultrasound literature, CT-specific reference data remain limited. CT is frequently performed for abdominal and pelvic indications, and the bladder is routinely visualized even when urinary tract evaluation is not the primary purpose of the examination. Fananapazir et al. provided important CT-based reference data and found that bladder volume was the dominant predictor of CT-measured BWT, whereas age and gender contributed minimally [5]. However, formal CT volumetry is not routinely performed during daily interpretation, limiting the immediate clinical utility of volume-based thresholds.

For routine CT interpretation, a single diameter measurement may be more practical than formal bladder volumetry. The sagittal long-axis bladder diameter (SL), corresponding to the largest midsagittal luminal bladder diameter, can be obtained on sagittal reformatted CT images. Prior cross-sectional imaging research demonstrated a strong association between SL and bladder volume, supporting SL as a practical single-dimensional indicator of bladder distension [6]. The present study builds on that evidence by examining the SL-BWT relationship in female patients. Bladder volume was not measured in this cohort, so equivalence between SL-based and volume-based BWT assessment was not evaluated.

This issue is clinically relevant because incidental bladder wall thickening is common in routine CT interpretation and can trigger downstream evaluation. Byler et al. reported urothelial carcinoma in a subset of patients undergoing cystoscopy for incidental CT-detected bladder wall abnormalities [7], while other studies have reported variable malignancy yield for diffuse or focal thickening in the absence of a discrete mass [8,9]. Non-neoplastic entities, including cystitis, chronic inflammation, trabeculation, and interstitial cystitis/bladder pain syndrome, can also produce diffuse or focal wall thickening [10,11]. Thus, radiologists must interpret BWT in light of bladder distension and morphology rather than wall thickness alone.

Female patients represent a particularly important population for BWT assessment because urinary tract infections, overactive bladder, interstitial cystitis/bladder pain syndrome, pelvic floor dysfunction, and postoperative or gynecologic influences may all affect bladder imaging appearances. Recent ultrasound-focused systematic review data in women have emphasized persistent heterogeneity in BWT measurement methodology and limited diagnostic reliability when filling conditions are not standardized [12]. The present study intentionally focuses on female patients to provide CT-based data relevant to bladder assessment in this population.

This study evaluates CT-measured BWT as a function of SL in female patients without documented bladder disease or urinary symptoms. Categorical and continuous analyses describe the relationship between these measurements and explore whether accounting for age changes the association. The study does not establish a diagnostic threshold for bladder disease.

## Materials and methods

Study population

A total of 111 patients aged 16 years or older and recorded as female in the source clinical imaging dataset were included. Sex was categorized using the demographic sex field available in the source record. “Healthy” was defined as having no urinary tract symptoms at the time of presentation, no known chronic or inflammatory bladder disease or neurogenic bladder, no history of pelvic surgery or pelvic radiotherapy, no history of recurrent urinary tract infection, and no clinical suspicion of cystitis within the preceding month. Eligibility was determined by review of the available medical record, including documented clinical history. Patients younger than 16 years (n=2; ages 3 and 15 years) were excluded because age-related differences in pediatric bladder size and capacity could limit application of a common SL reference point. The resulting cohort ranged from 16 to 92 years of age (mean: 54.3 years; median: 55 years).

CT measurement technique

CT examinations were typical trauma-protocol studies of the abdomen and pelvis performed at a single academic center using Revolution or LightSpeed VCT scanners (GE HealthCare, Waukesha, USA) or SOMATOM scanners (Siemens Healthcare GmbH, Erlangen, Germany). Source axial images had a thickness of 0.9 mm and were reconstructed as 3-mm axial, sagittal, and coronal series with a reconstruction interval of 3 mm, according to institutional body-alignment protocols. All measurements were performed on the midsagittal reformatted images sent to the picture archiving and communication system (PACS). A soft-tissue abdominal window was used, with an approximate window width of 350-400 Hounsfield units (HU) and level of 40-50 HU. In-plane pixel spacing was approximately 0.6-0.9 mm, typically 0.7-0.8 mm, depending on the field of view and matrix size.

SL was measured as the greatest dimension of the bladder lumen confined by the inner bladder walls on the selected midsagittal image. BWT was measured at the anterior bladder wall at the point of contact with the SL line. The caliper was placed perpendicular to the wall, from the urine-inner wall interface to the outer wall-perivesical fat interface. All measurements were performed by a single board-certified abdominal radiologist with more than 20 years of experience in abdominal and pelvic imaging.

The anterior wall provided a consistent anatomical measurement site. Repeated blinded measurements were not performed, and neither intraobserver nor interobserver reproducibility was assessed. The reconstruction kernel was not available for this analysis. SL values recorded in centimeters were converted to millimeters for analysis and reporting. SL was selected as a practical indicator of bladder distension based on its previously demonstrated association with bladder volume [6]. Direct bladder volumetry and measurement time were not evaluated in this cohort. Representative CT measurement examples are shown in Figures 1A-1H.

**Figure 1:**
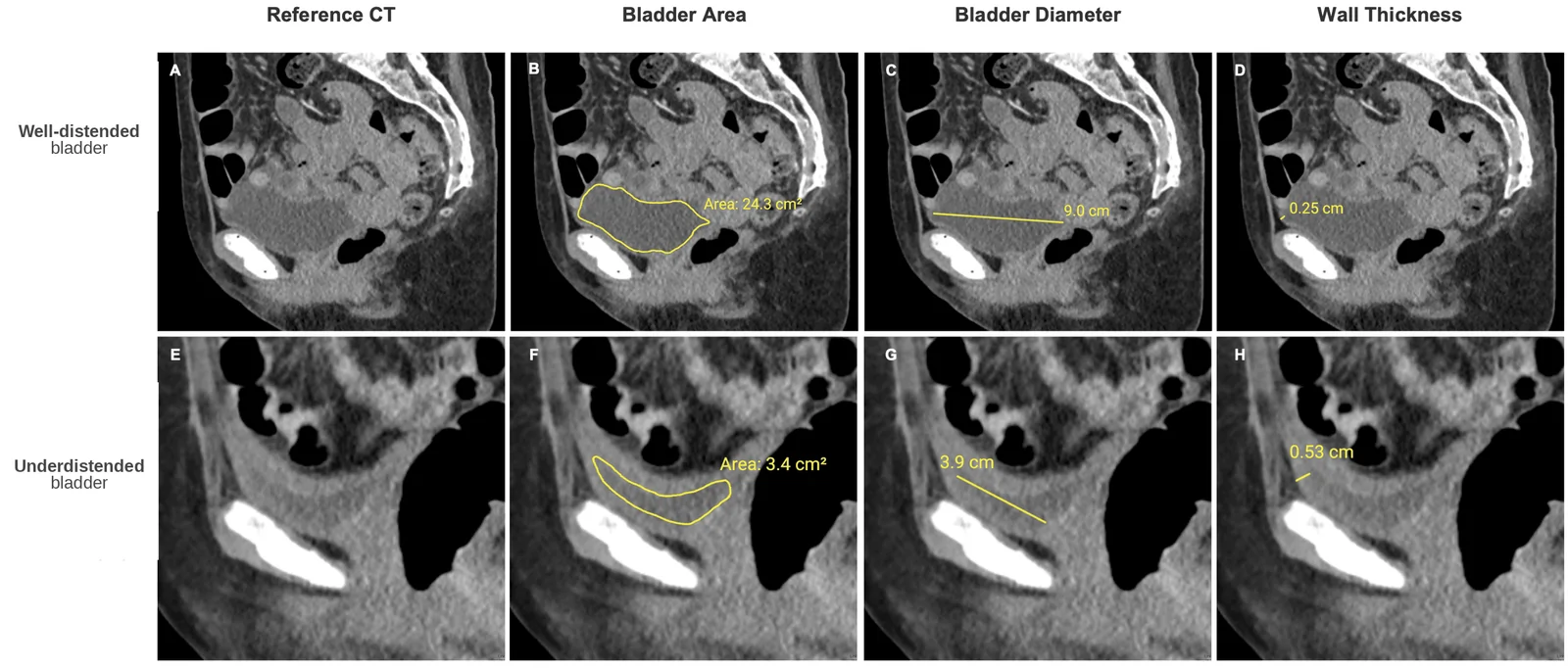
Representative sagittal CT examples of bladder distension and measurement technique. Panels A-D show a well-distended bladder, and panels E-H show an underdistended bladder. Panels A and E show reference sagittal CT images. Panels B and F demonstrate bladder contour/area tracing for visual assessment of bladder distension. Panels C and G demonstrate measurement of sagittal long-axis bladder diameter (SL), defined as the largest midsagittal bladder diameter. Panels D and H demonstrate bladder wall thickness (BWT) measurement. The source overlays display values in centimeters: 9.0 and 3.9 cm correspond to 90 and 39 mm for SL, while 0.25 and 0.53 cm correspond to 2.5 and 5.3 mm for BWT. Yellow annotations indicate the corresponding measurement overlays. Reduced bladder distension is associated with a smaller SL and a thicker-appearing bladder wall. CT, computed tomography.

SL distribution was summarized in 10-mm bins from 10-<20 mm through 180-<190 mm. For categorical analysis of BWT, SL was grouped as follows: 10-<30 mm, 30-<50 mm, 50-<70 mm, 70-<90 mm, and ≥90 mm. These categories were selected to preserve adequate sample size while separating small, intermediate, and larger bladder distension levels.

Statistical analysis

Descriptive statistics summarized BWT and SL. SL frequency was displayed as a 10-mm binned distribution. One-way analysis of variance (ANOVA) tested for differences in BWT across the five SL categories, and Tukey's honestly significant difference (HSD) post hoc testing evaluated pairwise differences. Pearson correlation assessed the linear relationship between SL and BWT. Simple linear regression and log-log power-law regression models were fitted with BWT as the dependent variable and SL as the independent variable. For the power-law model, both variables were natural log-transformed before regression. Statistical significance was defined as p < 0.05.

Ethical analysis

This study was reviewed by the Augusta University Institutional Review Board (IRB; Committee A; reference no. 2326790-1) and was determined not to constitute human subjects research because it involved retrospective analysis of de-identified imaging data without direct patient contact, intervention, or access to protected health information. Formal IRB approval was therefore not required, and the requirement for informed consent was waived. The study was conducted in accordance with institutional policies for the responsible use of clinical data.

## Results

Study population and overall measurements

A total of 111 healthy female patients were included in the analysis. The age range was 16 to 92 years. Across the cohort, mean BWT was 3.50 mm (SD: 2.11), with a median of 3.0 mm and a range of 1 to 12 mm. Mean SL was 62.05 mm (SD: 26.73), with a median of 60.60 mm and a range of 12.0 to 189.6 mm.

Distribution of sagittal long-axis bladder diameter (SL)

The distribution of SL was concentrated in the intermediate range, with the highest frequencies in the 50-<60 mm and 60-<70 mm bins. The most common 10-mm interval was 60-<70 mm (n = 22), followed by 50-<60 mm (n = 18), 40-<50 mm (n = 16), 70-<80 mm (n = 14), and 80-<90 mm (n = 12). SL values ≥90 mm were uncommon, with only 10 patients in this range. The distribution is shown in Figure 2.

**Figure 2:**
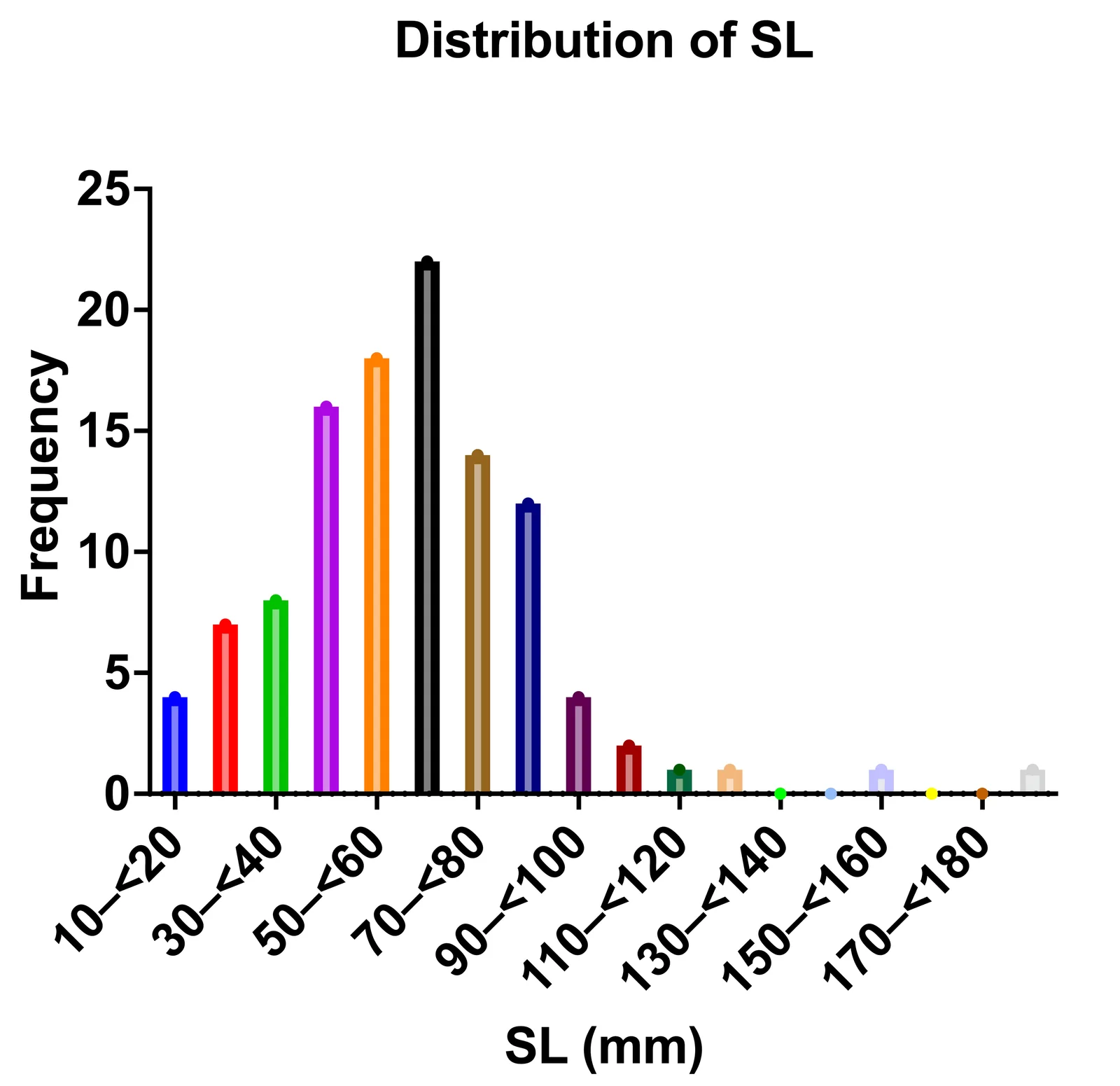
Distribution of sagittal long-axis bladder diameter in female patients. Histogram showing the frequency of sagittal long-axis bladder diameter (SL) across 10-mm intervals in the study cohort (n=111). Most patients clustered between 40 and 90 mm, with the highest frequency in the 60-<70 mm bin. Alternate x-axis labels are displayed for readability. Each bin includes the lower bound and excludes the upper bound.

Effect of SL on wall thickness

Mean BWT decreased progressively as SL increased. In the 10-<30 mm group (n=11), mean BWT was 6.00 mm (SD: 3.10). This decreased to 4.50 mm (SD: 2.23) in the 30-<50 mm group (n=24), 3.23 mm (SD: 1.70) in the 50-<70 mm group (n=40), 2.54 mm (SD: 0.86) in the 70-<90 mm group (n=26), and 1.90 mm (SD: 0.32) in the ≥90 mm group (n=10). One-way ANOVA demonstrated a significant effect of SL category on BWT (F=11.26; P<0.001). Category-specific values are summarized in Table 1.

**Table 1:** Bladder wall thickness by sagittal long-axis bladder diameter category. BWT, bladder wall thickness; CV, coefficient of variation; SL, sagittal long-axis bladder diameter; SD, standard deviation.

SL category (mm)	n	Mean BWT (mm)	SD (mm)	Median BWT (mm)	Range (mm)	CV (%)
10-<30	11	6.00	3.10	5.0	2-12	51.6
30-<50	24	4.50	2.23	4.0	2-9	49.5
50-<70	40	3.23	1.70	3.0	1-8	52.8
70-<90	26	2.54	0.86	2.0	1-4	33.9
≥90	10	1.90	0.32	2.0	1-2	16.6

Post hoc testing showed that the 10-<30 mm group had significantly greater BWT than the 50-<70 mm, 70-<90 mm, and ≥90 mm groups. The 30-<50 mm group had significantly greater BWT than the 70-<90 mm and ≥90 mm groups; its comparison with the 50-<70 mm group did not reach significance after Tukey adjustment (P=0.054). Differences among the three larger-SL groups were not statistically significant, supporting the interpretation that apparent wall thickening and measurement variability are most problematic at smaller SL values. The pairwise comparisons are detailed in Table 2.

**Table 2:** Tukey HSD pairwise comparisons of bladder wall thickness between sagittal long-axis bladder diameter categories. Mean BWT difference is reported as the smaller-SL category minus the larger-SL category. Positive values indicate greater BWT in the smaller-SL group. BWT, bladder wall thickness; CI, confidence interval; SL, sagittal long-axis bladder diameter; HSD, honestly significant difference.

Comparison	Mean BWT difference (mm)	95% CI (mm)	Adjusted P-value
10-<30 vs 30-<50	1.50	-0.32 to 3.32	0.155
10-<30 vs 50-<70	2.78	1.08 to 4.47	0.0001
10-<30 vs 70-<90	3.46	1.67 to 5.26	< 0.001
10-<30 vs ≥90	4.10	1.92 to 6.28	< 0.001
30-<50 vs 50-<70	1.28	-0.01 to 2.56	0.054
30-<50 vs 70-<90	1.96	0.55 to 3.37	0.0018
30-<50 vs ≥90	2.60	0.72 to 4.48	0.0019
50-<70 vs 70-<90	0.69	-0.57 to 1.94	0.554
50-<70 vs ≥90	1.33	-0.44 to 3.09	0.234
70-<90 vs ≥90	0.64	-1.22 to 2.49	0.875

Continuous relationship between SL and wall thickness

SL demonstrated a moderate inverse correlation with BWT (Pearson r=-0.51; P<0.001). Simple linear regression yielded BWT=-0.0398 × SL+5.964 (R²=0.255), indicating that each additional millimeter of SL was associated with an approximately 0.04-mm decrease in measured wall thickness. The fitted linear relationship, including 95% confidence and prediction intervals, is shown in Figure 3.

**Figure 3:**
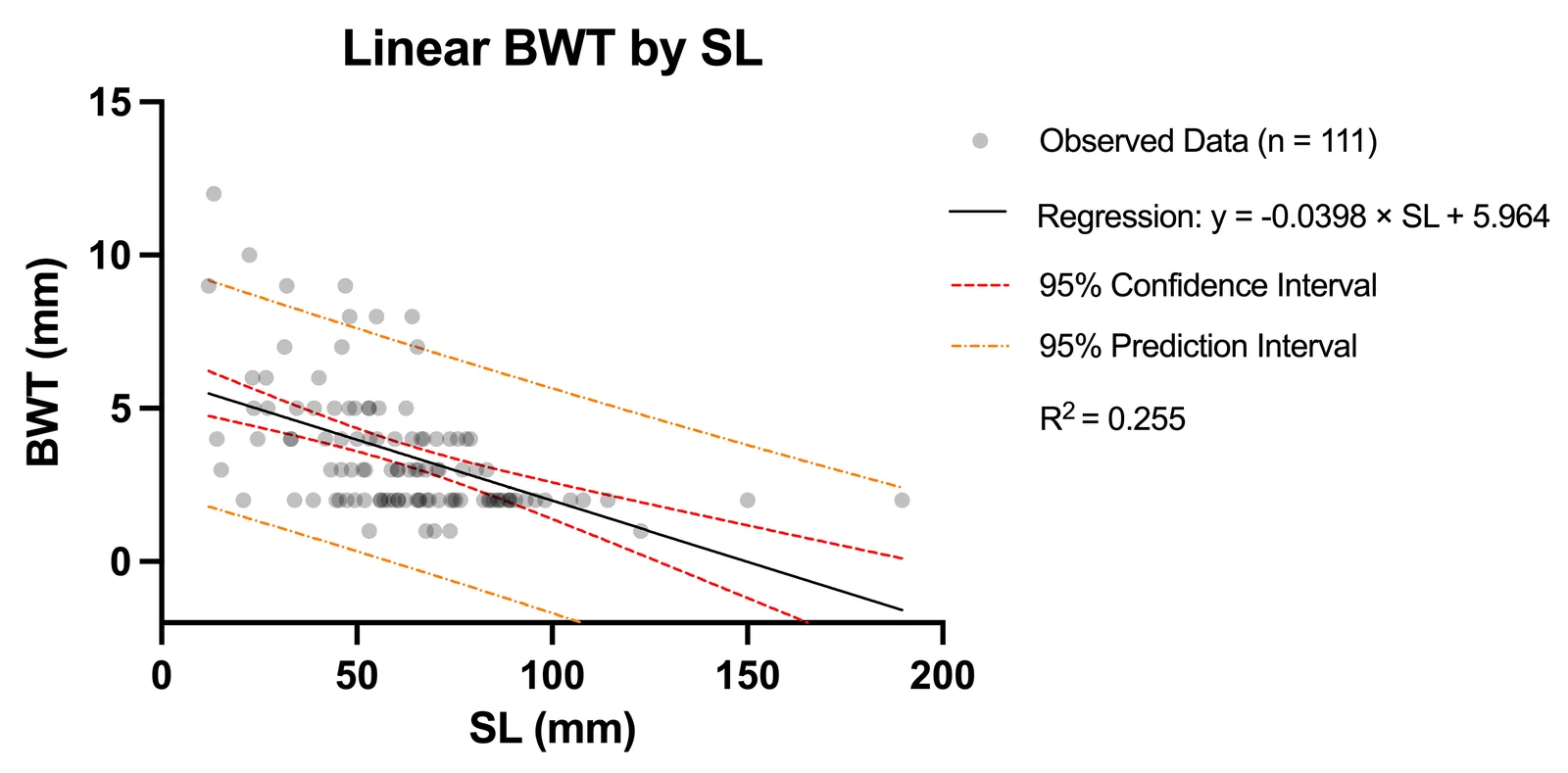
Linear relationship between the bladder wall thickness and sagittal long-axis bladder diameter. Scatter plot showing bladder wall thickness (BWT) versus sagittal long-axis bladder diameter (SL). The fitted simple linear regression line is shown with 95% confidence intervals for the mean estimate and 95% prediction intervals for individual observations. The model was BWT=-0.0398 × SL+5.964 (R²=0.255; n=111).

Given the expected nonlinear relationship between bladder distension and wall thickness, a log-log power-law model was also evaluated. The power-law model improved the explained variance to R²=0.302 and yielded BWT=35.87 × SL⁻⁰^·^⁶¹⁵. The negative exponent indicates that BWT decreases most sharply at smaller SL values and gradually plateaus as the bladder becomes more distended. At an SL of 70 mm, the model predicts a BWT of approximately 2.63 mm. The power-law relationship is shown in Figure 4.

**Figure 4:**
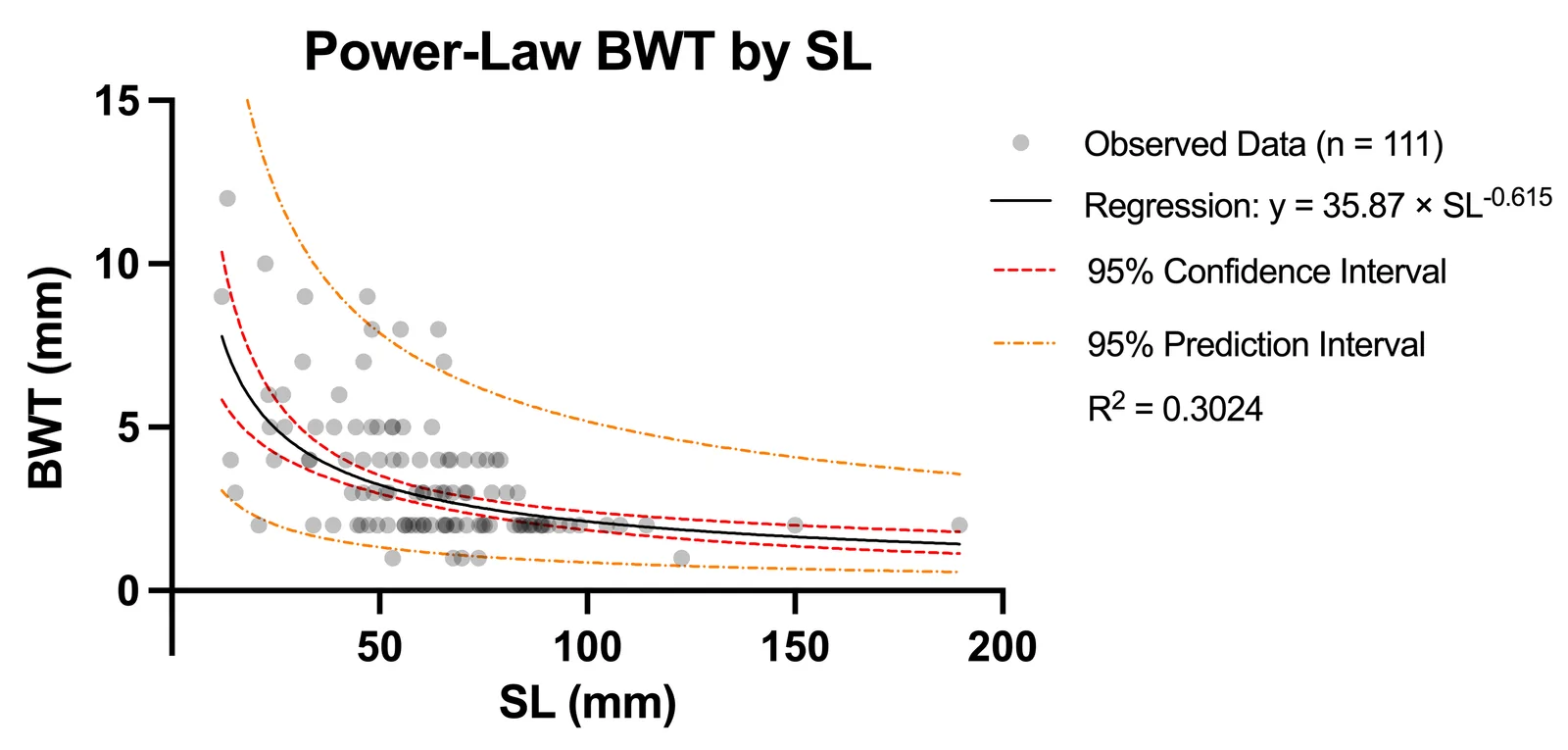
Power-law relationship between the bladder wall thickness and sagittal long-axis bladder diameter. Scatter plot showing bladder wall thickness (BWT) versus sagittal long-axis bladder diameter (SL) with a log-log power-law regression model. The curve demonstrates a nonlinear inverse relationship, with BWT decreasing most sharply at smaller SL values and gradually plateauing as SL increases. The model was BWT=35.87 × SL⁻⁰^·^⁶¹⁵ (R²=0.302; n=111).

## Discussion

This study found an inverse association between CT-measured BWT and SL in female patients without documented bladder disease or urinary symptoms. Smaller SL values were associated with thicker and more variable bladder walls, whereas larger SL values were associated with thinner walls and less between-patient variation. The study extends prior evidence relating SL to bladder volume [6] by describing how BWT varies with this practical indicator of distension in female patients. Recording SL alongside BWT may help describe wall thickness in relation to bladder distension, although the present findings do not distinguish normal from diseased bladder walls.

Comparison with prior CT literature

The closest CT comparison is the study by Fananapazir et al., which evaluated 419 trauma patients with normal abdominal and pelvic CT examinations [5]. Bladder volume was measured using both an ellipsoid formula and an automated technique. Volume accounted for most of the explanatory contribution to their models, while age and sex added little. The present findings are consistent with their central observation that expected BWT depends on bladder distension. Our analysis extends this approach to an SL-based description in a clinically selected female cohort.

The choice of SL is supported by Nelson et al., who reported a strong correlation between SL and manually traced bladder volume (r = 0.92) in 80 individuals without urinary tract pathology [6]. This provides an empirical basis for using SL as a practical indicator of bladder distension. The current analysis addresses the complementary question of how CT-measured BWT varies with SL in female patients.

Direct volumetry was not performed in this cohort, so the relative performance of SL-based and volume-based BWT models remains to be compared within the same patients. Such a comparison could determine whether incorporating volume or bladder shape materially improves BWT prediction beyond SL alone. Reader measurement time and reproducibility would also help establish the practical value of either approach.

Comparison with ultrasound literature

The direction of the association is consistent with the ultrasound literature. Jequier and Rousseau described thinner bladder walls with greater filling in children, and Hakenberg et al. reported a weak inverse association between bladder volume and BWT in adults [3,4]. Hakenberg et al. reported a mean BWT of approximately 3 mm in healthy adult women [4]. Although this is broadly similar to the values observed in the intermediate and larger SL categories here, it should not be treated as confirmation of a common normal threshold. The studies differed in patient selection, filling conditions, measurement technique, and imaging modality. Pediatric observations support the general filling-dependent relationship but do not validate the numerical SL categories used in this cohort.

Technical validation studies help explain why numerical thresholds cannot simply be transferred between methods. Asfour et al. compared ultrasound measurements with direct caliper measurements of 25 cadaveric bladder dome specimens and found that accuracy depended on ultrasound frequency and the placement of measurement boundaries [13]. Panayi et al. reported better interobserver agreement with a transvaginal approach than with transperineal or transabdominal approaches [14]. CT measurement of the visible full wall at one anterior midsagittal site therefore requires its own validation. This distinction also matters when comparing BWT with ultrasound measurements restricted to the detrusor layer [1,15]. Using the same units does not make these measurements interchangeable.

The clinical interpretation of BWT in women has also become more cautious as evidence has accumulated. The earlier review by Oelke et al. described promising associations between ultrasound-derived thickness and detrusor overactivity, but noted substantial variation in technique and proposed thresholds [1]. The 2026 systematic review and meta-analysis by Lemmon et al. reviewed eight studies, included six in the quantitative meta-analysis, and concluded that ultrasound BWT could not replace urodynamics for diagnosing detrusor overactivity [12]. This distinction between a group-level association and diagnostic discrimination is directly relevant to the present findings. An association between SL and BWT in patients without recognized bladder disease does not show that SL-adjusted thickness can identify functional bladder disorders. The present results provide descriptive data for that future question.

Distension, variability, and interpretation of the models

The observed pattern is compatible with the filling-dependent changes described in previous ultrasound and CT studies [3-5]. A relatively underdistended bladder may have a thicker-appearing wall, while greater distension is associated with a thinner appearance. However, each patient in this study was measured at a single examination. The data therefore demonstrate an association across patients and do not directly measure how an individual bladder changes during filling. The power-law curve provides a descriptive representation of a steeper decline at smaller SL values followed by a more gradual change; the fitted shape should not be taken as direct evidence of a particular tissue mechanism.

Lower standard deviations and coefficients of variation in the larger SL categories indicate less dispersion between patients in this sample. They do not establish greater measurement reliability or reproducibility. Those properties require repeated measurements, ideally by independent readers. The ICI-RS report emphasized standardization of measurement conditions and technique for bladder and detrusor wall thickness assessment [2]. The present use of a consistent anterior-wall location addresses one source of procedural variation, but the single-reader design cannot determine whether the same results would be obtained by other radiologists.

The SL categories should also remain descriptive. The value of 70 mm is a boundary between the study categories, not a cutoff derived from diagnostic testing. The cohort contained no disease comparison group from which sensitivity, specificity, or a clinically useful decision threshold could be established. In addition, the linear and log-log models use different outcome scales, so their R² values alone do not establish which model predicts BWT more accurately in millimeters. Confidence intervals describe uncertainty around the fitted relationship, whereas prediction intervals describe the wider uncertainty for an individual observation. Neither interval provides a validated boundary between normal and abnormal bladder walls.

Age and robustness of the association

The added analyses did not detect a statistically significant association of age with BWT or SL, and adjustment for age did not materially change the SL coefficient. Restriction to patients aged 18 years or older also retained the inverse association. These findings address age as one potential source of confounding in this cohort; they do not establish that age has no effect on bladder morphology or exclude smaller, nonlinear, or age-dependent associations. Fananapazir et al. likewise found that age contributed relatively little to CT-based BWT models once bladder volume was considered [5], although the methods and populations differ. Our analyses do not establish age-specific reference ranges.

The influence analysis also places the regression findings in context. The largest-SL observation had substantial leverage and changed the magnitude of the fitted linear slope, but omitting it did not reverse or eliminate the inverse association. The power-law exponent and illustrative fitted value at 70 mm changed less. These sensitivity analyses support the direction of the association while showing why precise original-scale predictions at the extremes require caution. Agreement with the source worksheet is not confirmation of imaging accuracy; the original CT measurements were not repeated.

Clinical interpretation and incidental bladder wall thickening

The potential clinical use of SL is to document bladder distension when BWT is described on an existing CT examination. Evans et al. documented the broader frequency of incidental findings on emergency department CT, although their review was not specific to bladder abnormalities [16]. For bladder findings, recording SL could provide a consistent description of size alongside the location and morphology of apparent thickening. The present study did not test whether this changes diagnostic confidence, report wording, referrals, or the use of additional investigations.

Studies of incidental bladder wall abnormalities show why a distension measurement should not be used to dismiss a finding. McPartlin et al. found no malignancy among patients with incidental diffuse or focal thickening in their small series, while cancers occurred in patients with focal mass lesions [8]. In contrast, Byler et al. identified urothelial carcinoma among patients evaluated for incidental bladder abnormalities, including diffuse thickening [7]. Song et al. found malignancy in 11 of 167 patients undergoing cystoscopy for incidentally detected BWT [9]. These selected referral cohorts differ from the clinically screened reference population studied here. Differences in selection and classification may contribute to the divergent findings, and our data cannot resolve the appropriate workup of incidental thickening.

The appropriate interpretation is therefore limited: SL may describe one factor contributing to the appearance of the wall. It has not been shown to determine whether that appearance is benign. Assessment still requires attention to focal or mass-like morphology and to the clinical presentation, because neoplastic and nonneoplastic lesions may overlap on imaging [10]. A small SL should not be used to explain away a suspicious lesion, and a larger SL does not establish that diffuse thickening is pathological. Clinical outcome studies are needed before SL can be incorporated into decisions about further investigation.

Relevance to inflammatory bladder disease

Disease-specific CT studies provide another reason to avoid interpreting BWT solely as a consequence of distension. Yu et al. evaluated 182 patients with cystoscopy-proven interstitial cystitis/bladder pain syndrome and found that focal or diffuse wall thickening was associated with more severe clinical and histopathological abnormalities, including smaller bladder capacity and greater inflammatory changes [11]. That population differs fundamentally from the present cohort, which excluded recognized bladder disease and relevant symptoms. The two studies address complementary aspects of the same imaging appearance: variation associated with bladder size in a reference population and thickening associated with established disease. A future study including symptomatic and asymptomatic patients would be needed to determine whether SL contributes useful information beyond morphology and clinical findings.

Strengths and limitations

The intentional focus on female patients is a strength because the study was designed to inform CT assessment of the bladder in this population. It provides data directly relevant to that objective without combining female and male observations. The cohort was clinically selected using exclusions for urinary tract symptoms, recent suspected cystitis, recurrent urinary tract infection, known bladder disease, neurogenic bladder, prior pelvic surgery, and pelvic radiotherapy. A consistent anterior midsagittal measurement site was used by a board-certified abdominal radiologist with more than 20 years of experience in abdominal and pelvic imaging. Evaluating both categorical and continuous relationships also allowed the observed pattern to be described without relying on a single proposed cutoff.

Several limitations affect interpretation. The retrospective, single-center design may limit generalizability to other clinical settings, and eligibility depended on the completeness of the available clinical records. The absence of documented disease does not exclude unrecognized or subclinical pathology. Regional differences in BWT were not assessed. Although age-adjusted analyses and an age ≥18-year sensitivity analysis were added, the study was not designed to establish age-specific reference ranges or exclude smaller or nonlinear age-related effects. The largest-SL category contained few patients, and the most extreme observation influenced the linear slope. No extreme observation was removed from the primary cohort.

Thin-wall measurements must be considered in the context of CT spatial resolution. BWT values of 1-3 mm measured on 3-mm sagittal reformats may be affected by partial-volume averaging, wall orientation, and boundary placement. Through-plane slice thickness and in-plane pixel spacing describe different aspects of image sampling; neither the 0.9-mm source-image thickness nor submillimeter pixel spacing eliminates these effects. The reconstruction interval, approximate window settings, and pixel-spacing range are reported in Methods to facilitate replication, although the reconstruction kernel was unavailable. A consistent site and one experienced reader provide procedural consistency, but do not demonstrate accuracy or intraobserver or interobserver reproducibility. No repeated or independent-reader measurements were performed.

Although prior work supports SL as an indicator of bladder distension [6], direct volumetry was not performed here, so the study could not compare SL-based and volume-based BWT models or separate the contribution of bladder shape to residual variation. The cohort also lacked a disease comparison group. Conventional ANOVA, Tukey testing, and plotted regression intervals have distributional and variance assumptions that may be imperfect when group dispersions differ. HC3 inference in the added regression analyses addresses unequal variance for coefficient inference, but does not validate the individual prediction intervals or diagnostic performance. The models were developed and evaluated in the same cohort, so their performance in new patients remains uncertain. These limitations are particularly relevant to any proposed use outside the study population, including patients with active cystitis, interstitial cystitis/bladder pain syndrome, neurogenic bladder, chronic outlet obstruction, prior pelvic radiation, or altered bladder anatomy.

Future directions

Further work should evaluate the reproducibility and usefulness of SL-based BWT assessment in independent female cohorts. Repeated measurements by multiple readers would distinguish measurement reproducibility from between-patient variation. Comparing SL-based and volume-based BWT models within the same patients would establish whether direct volumetry adds useful predictive information. A study including patients with bladder disease could then assess whether SL improves interpretation beyond clinical information and bladder morphology, including its effects on diagnostic accuracy, reporting, and referral.

## Conclusions

CT-measured BWT decreased with increasing SL in female patients without documented bladder disease or urinary symptoms. The findings extend prior evidence supporting SL as a practical indicator of bladder distension and provide data specific to CT bladder assessment in female patients. Recording SL alongside BWT may help describe wall thickness in relation to distension. The descriptive categories and fitted relationships do not establish diagnostic thresholds or determine the need for further investigation. Independent studies should evaluate reader reproducibility, compare SL-based and volume-based BWT models, and assess diagnostic usefulness in patients with bladder disease.
